# Intra-pocket ultrasound-guided axillary vein puncture vs. cephalic vein cutdown for cardiac electronic device implantation: the ACCESS trial

**DOI:** 10.1093/eurheartj/ehad629

**Published:** 2023-10-13

**Authors:** Paul Charles, Geoffroy Ditac, Mathieu Montoy, Thibaut Thenard, Pierre-Yves Courand, Pierre Lantelme, Brahim Harbaoui, Samir Fareh

**Affiliations:** Fédération de Cardiologie, Hôpital de la Croix Rousse et Hôpital Lyon Sud, Hospices Civils de Lyon, 103 Grande Rue de la Croix-Rousse, 69004 Lyon, France; Fédération de Cardiologie, Hôpital de la Croix Rousse et Hôpital Lyon Sud, Hospices Civils de Lyon, 103 Grande Rue de la Croix-Rousse, 69004 Lyon, France; Fédération de Cardiologie, Hôpital de la Croix Rousse et Hôpital Lyon Sud, Hospices Civils de Lyon, 103 Grande Rue de la Croix-Rousse, 69004 Lyon, France; Fédération de Cardiologie, Hôpital de la Croix Rousse et Hôpital Lyon Sud, Hospices Civils de Lyon, 103 Grande Rue de la Croix-Rousse, 69004 Lyon, France; Fédération de Cardiologie, Hôpital de la Croix Rousse et Hôpital Lyon Sud, Hospices Civils de Lyon, 103 Grande Rue de la Croix-Rousse, 69004 Lyon, France; Université de Lyon, CREATIS UMR5220, INSERM U1044, INSA Lyon, 7 avenue Jean Capelle, 69621 Villeurbanne Cedex, Lyon, France; Fédération de Cardiologie, Hôpital de la Croix Rousse et Hôpital Lyon Sud, Hospices Civils de Lyon, 103 Grande Rue de la Croix-Rousse, 69004 Lyon, France; Université de Lyon, CREATIS UMR5220, INSERM U1044, INSA Lyon, 7 avenue Jean Capelle, 69621 Villeurbanne Cedex, Lyon, France; Fédération de Cardiologie, Hôpital de la Croix Rousse et Hôpital Lyon Sud, Hospices Civils de Lyon, 103 Grande Rue de la Croix-Rousse, 69004 Lyon, France; Université de Lyon, CREATIS UMR5220, INSERM U1044, INSA Lyon, 7 avenue Jean Capelle, 69621 Villeurbanne Cedex, Lyon, France; Fédération de Cardiologie, Hôpital de la Croix Rousse et Hôpital Lyon Sud, Hospices Civils de Lyon, 103 Grande Rue de la Croix-Rousse, 69004 Lyon, France

**Keywords:** Randomized clinical trial, Ultrasound guidance, Axillary vein, Cephalic vein, Pacemaker, Implantable cardioverter-defibrillator

## Abstract

**Background and Aims:**

Intra-pocket ultrasound-guided axillary vein puncture (IPUS-AVP) for venous access in implantation of transvenous cardiac implantable electronic devices (CIED) is uncommon due to the lack of clinical evidence supporting this technique. This study investigated the efficacy and early complications of IPUS-AVP compared to the standard method using cephalic vein cutdown (CVC) for CIED implantation.

**Methods:**

ACCESS was an investigator-led, interventional, randomized (1:1 ratio), monocentric, controlled superiority trial. A total of 200 patients undergoing CIED implantation were randomized to IPUS-AVP (*n* = 101) or CVC (*n* = 99) as a first assigned route. The primary endpoint was the success rate of insertion of all leads using the first assigned venous access technique. The secondary endpoints were time to venous access, total procedure duration, fluoroscopy time, X-ray exposure, and complications. Complications were monitored during a follow-up period of three months after procedure.

**Results:**

IPUS-AVP was significantly superior to CVC for the primary endpoint with 100 (99.0%) vs. 86 (86.9%) procedural successes (*P* = .001). Cephalic vein cutdown followed by subclavian vein puncture was successful in a total of 95 (96.0%) patients, *P* = .21 vs. IPUS-AVP. All secondary endpoints were also significantly improved in the IPUS-AVP group with reduction in time to venous access [3.4 vs. 10.6 min, geometric mean ratio (GMR) 0.32 (95% confidence interval, CI, 0.28–0.36), *P* < .001], total procedure duration [33.8 vs. 46.9 min, GMR 0.72 (95% CI 0.67–0.78), *P* < .001], fluoroscopy time [2.4 vs. 3.3 min, GMR 0.74 (95% CI 0.63–0.86), *P* < .001], and X-ray exposure [1083 vs. 1423 mGy.cm², GMR 0.76 (95% CI 0.62–0.93), *P* = .009]. There was no significant difference in complication rates between groups (*P* = .68).

**Conclusions:**

IPUS-AVP is superior to CVC in terms of success rate, time to venous access, procedure duration, and radiation exposure. Complication rates were similar between the two groups. Intra-pocket ultrasound-guided axillary vein puncture should be a recommended venous access technique for CIED implantation.

## Structured Graphical Abstract

**Structured Graphical Abstract:**
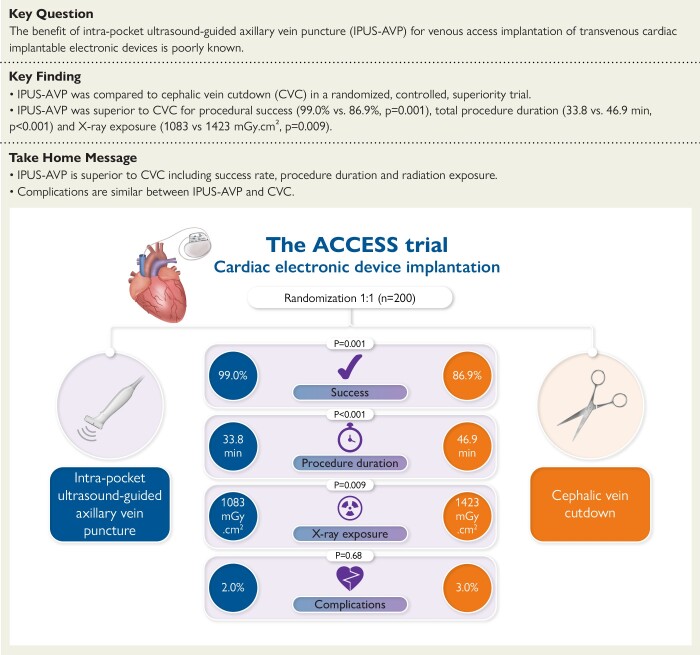
Efficacy and safety of intra-pocket ultrasound-guided axillary vein access vs. cephalic vein cutdown for implantation of cardiac electronic devices. CVC, cephalic vein cutdown; IPUS-AVP, intra-pocket ultrasound-guided axillary vein puncture.

**See the editorial comment for this article ‘Different techniques of venous access for CIEDs: advantages and disadvantages’, by M. Brignole and J.-C. Deharo, https://doi.org/10.1093/eurheartj/ehad749.**

## Introduction

Venous access is the first and challenging step in lead insertion during implantation of transvenous cardiac implantable electronic devices (CIED) such as pacemakers and implantable cardioverter-defibrillators (ICD). Cephalic vein cutdown (CVC), subclavian vein puncture (SVP), and axillary vein puncture (AVP) are the three principal techniques for venous access.^1,2^ SVP is a fast technique with a high success rate.^3^ However, it presents higher risks of pneumothorax and lead dysfunction by crush syndrome compared to the two other techniques.^1^ It is therefore no longer recommended for first-line venous access.^2,4^ Cephalic vein cutdown is the most commonly used technique in Europe.^5^ It causes fewer complications but is more time consuming and the cephalic vein is sometimes impossible to cannulate, thereby leading to SVP as second-line technique for venous access.^6–11^ More recently, fluoroscopy-guided AVP, with or without venography, has also been studied.^9,11–14^ This vascular access technique would offer the combined advantages of the two previous techniques, although complications related to the puncture are not fully prevented.

The extra-thoracic position of the axillary vein allows the use of ultrasound (US) guidance, which is recommended in critical care and anaesthesiology to improve success and reduce complications of central venous access.^15–17^ For CIED implantation, US-guided vascular access is mentioned as promising in recent guidelines, but there is not enough clinical evidence to make a recommendation.^2,4^ Only two small randomized trials have investigated US-guided AVP using a percutaneous approach.^18–20^ These studies have shown a good overall success of the technique but a higher risk of failure in case of deep vein or elevated body mass index (BMI). Ultrasound guidance performed through the open incision (intra-pocket) with a small-footprint probe could overcome these limitations. This approach has only been described in a few observational studies.^21–23^ We propose to investigate this intra-pocket US-guided AVP technique (IPUS-AVP) for CIED implantation.

The objective of the randomized controlled ACCESS trial (intra-pocket US-guided axillary vein access vs. CVC for implantation of cardiac electronic devices) was to evaluate the efficacy and the early complications of IPUS-AVP vs. CVC for CIED implantation.

## Materials and methods

### Study design

ACCESS was an investigator-led, interventional, prospective, single centre, randomized, and controlled superiority trial. Patients were randomized in a 1:1 ratio to receive vascular access with either IPUS-AVP (investigational arm) or CVC (control arm) as a first assigned route. Randomization was stratified according to the number of leads to be implanted (single- or dual-chamber device).

The trial was funded by the Hospices Civils de Lyon and was conducted in accordance with the Declaration of Helsinki and the International Council for Harmonisation Good Clinical Practice Guidelines. Data collection was managed and monitored by an independent team from the Department of Clinical Research and Innovation of the Hospices Civils de Lyon. In view of the low risk of serious adverse events associated with this research, no other committee was required for this study. The protocol was approved by the local ethic committee (CPP Sud-Ouest et Outre-mer, 31 March 2020) and was registered at ClinicalTrials.gov with number NCT04649788.

### Trial population

The patients enrolled were adults (≥18 years of age) with an indication for permanent transvenous pacemaker or ICD, either single chamber or dual chamber. Patients were excluded if they had a history of previously implanted endocardial lead, an indication for cardiac resynchronization therapy, impossible superior central venous access [superior vena cava (SVC) syndrome, thrombosis] or need for vein preservation (e.g. for haemodialysis). Patient’s demographics, clinical characteristics, and laboratory tests were recorded at baseline. All patients provided written informed consent prior to the intervention.

### Operators and procedure

The procedures were performed in a tertiary care centre by three cardiac electrophysiologists. Each operator had greater than 5 years of experience in CIED implantation with CVC and SVP, and greater than 1 year of experience with IPUS-AVP (over 400 IPUS-AVP procedures in total). Fellows and trainees could participate in the intervention but never as a primary operator.

Procedures could be urgent (unplanned admission) or scheduled. Peri-procedural antithrombotic treatment management was left to the operator’s discretion. Usually, in our centre, vitamin K antagonists are maintained with an International Normalized Ratio (INR) in the therapeutic range. According to guidelines, direct oral anticoagulants are generally stopped the day before the procedure (12 or 24 h, depending on the treatment) and resumed the day after, or sometimes two days after in case of high bleeding risk (elderly patients, chronic kidney disease, combination with antiplatelet therapy) in patients without previous embolic events.^4,24^ Antibiotic prophylaxis was administered to each patient before the procedure according to hospital protocol. The side of implantation (left or right) was chosen according to the preference of the operator and/or the patient but the configuration of the operating room facilitated an operation on the left side. The infraclavicular region was cleaned, and sterile surgical drapes were applied.

#### Intra-pocket ultrasound-guided axillary vein puncture group

**Figure 1:**
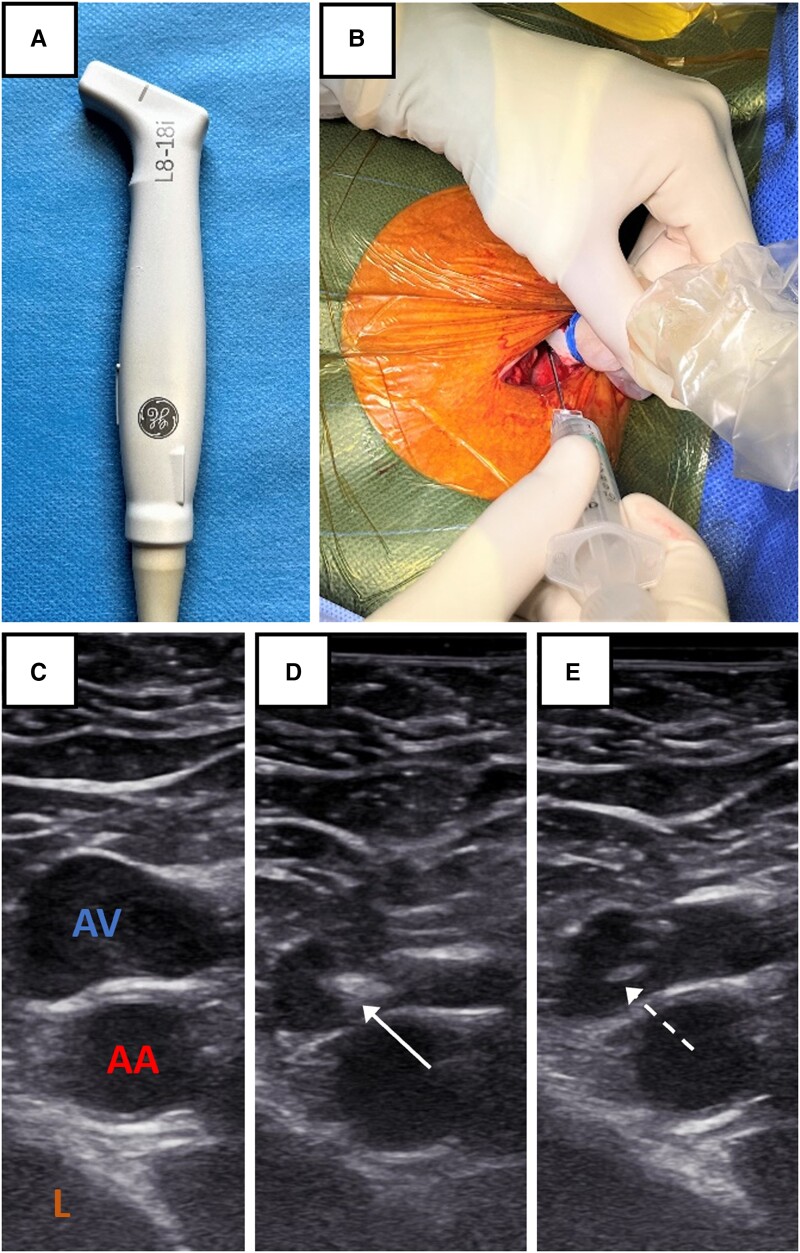
Illustration of the intra-pocket ultrasound-guided axillary vein puncture technique. (A) ‘Hockey stick’ ultrasound transducer. (B) Transducer and needle configuration for ultrasound-guided axillary venous puncture. The operator is on the left side of the patient. The transducer is held with the left hand and the needle with the right hand. (C) Cross-sectional view of axillary vein (AV) and artery (AA). The lung (L) is also visualized. (D) Needle tip (solid white arrow) is seen advancing in the axillary vein. (E) Needle tip (dashed white arrow) is seen in the lumen of the vein.

In the IPUS-AVP group, after preparation of the sterile field, an L-shaped ‘hockey stick’ small-footprint US transducer (L8–18i probe, General Electric Healthcare, Boston, MA, USA), linked to an US scanner (Vivid T8, General Electric Healthcare), was handed to the operator in a sterile way and placed in a sterile sheath with gel inside. The US probe is presented in *Figure 1A*.

After local anaesthesia, an incision was made 1 cm medial to the deltopectoral groove, subcutaneous tissues were dissected down to the pectoral muscle, and a subcutaneous pocket was created for the generator. The US probe was then placed in contact with the pectoral muscle, perpendicularly to the axis of the axillary vessels. Saline was used as the US contact medium. Venous access was performed through IPUS-AVP and Seldinger technique with an 18G needle attached to a syringe (*Figure 1B*). Vein and artery were visualized in transverse view and differentiated with pulse activity observation, compression, or colour Doppler imaging (*Figure 1C*). While gently aspirating on the syringe, the needle was advanced towards the vein (*Figure 1D* and *E*). The tip of the needle was followed with US until cannulation was successful with venous blood return in the syringe.

After successful puncture, a 0.035″ J-tipped guidewire was inserted and advanced to the SVC under fluoroscopy guidance. A standard tear-away sheath according to the size of the lead was inserted over the guidewire. If multiple leads were implanted, the operator was required to perform a distinct puncture for the second sheath and not use a retained guidewire technique. After all guidewires were inserted into the vein and visualized in the SVC under fluoroscopy guidance, the US probe was removed from the sterile field. A video illustrating the technique is available as *Supplementary data online* (see Supplementary data online, *Video S1*).

In case of failure with IPUS-AVP, the protocol specified that CVC should be performed in the second line, and then an SVP in the last intention.

#### Cephalic vein cutdown group

In the CVC group, an incision was made in the deltopectoral groove and subcutaneous tissues were dissected until the cephalic vein was identified. The vein was exposed and separated from its tissue attachments over approximately 2 cm before being cannulated to introduce the guidewire, which was positioned in the SVC under fluoroscopy guidance. If two leads were required, a second guidewire was introduced in the cephalic vein.

In case of failure with CVC, the protocol specified that SVP should be performed in the second line, and then an IPUS-AVP in the last intention.

#### Common parts of the procedure

In each group, in case of difficulties with the vascular access, operators had the option of using a hydrophilic guidewire, intravenous volume expansion, Trendelenburg position, or venous angiography.

After venous access was achieved, procedures were identical in both groups. Lead implantation was guided by fluoroscopy (Cios Alpha mobile C-arm, Siemens, Munich, Germany). The fluoroscopic system was configured to low-dose mode with low frame rates (5 frames/s) and proper collimation. Endocardial active or passive fixation leads were used in all patients. Atrial leads were implanted in the right atrial appendage, and right ventricle leads were positioned on the interventricular septum or at the right ventricular apex. The generator was placed in a pre-pectoral subcutaneous pocket. Skin was closed with absorbable thread. After dressing was applied, a compressive bandage (with roll and tape) was placed immediately and left until the following day.

After the procedure, the CIED pocket and the suture were examined daily until discharge. A chest X-ray was systematically performed within 24 h of the procedure to check for stability of the lead positioning and the absence of pneumothorax. Cardiac implantable electronic devices control was performed post-implantation and before patient discharge.

Each patient underwent an 8-day and a 3-month post-operative follow-up consultation with an electrophysiologist for pocket skin examination, electrocardiogram recording, and CIED interrogation.

### Endpoints and adverse events

The primary endpoint of this study was the success of insertion of all leads using the first assigned venous access technique (i.e. IPUS-AVP or CVC). Concerning dual-chamber CIED, if different venous accesses were required, the primary endpoint was not met. The decision to change the vascular access technique was at the discretion of the operator. There was no limit in the duration of the procedure or in the number of attempts, to reflect daily medical practice.

The secondary endpoints were time to venous access (from skin incision to presence of all required guidewires in the SVC), time from venous access to skin closure, total procedure duration (from skin incision to skin closure), fluoroscopy time, X-ray exposure (dose area product), and complications. Secondary endpoints were assessed for the entire strategy (i.e. all the vascular access techniques, if required) leading to a successful implantation of all leads.

Complications were monitored for up to 3 months after the intervention and were defined as: brachial plexus palsy; major pocket haematoma requiring evacuation or transfusion or prolongation of hospitalization; pneumothorax; haemothorax; pericardial effusion and tamponade; lead dislodgement or malfunction requiring revision; deep vein thrombosis; device infection requiring extraction. Electrical parameters of CIED including sensing, impedance, and pacing threshold were recorded at the end of implantation and at each follow-up visit.

### Statistical analysis

The primary hypothesis was that IPUS-AVP would be superior to CVC for the primary endpoint. Primary and secondary endpoints were tested for superiority according to the intention-to-treat (ITT) principle. Two groups were considered for the main analysis: IPUS-AVP and CVC. Different sensitivity analyses were performed and specifically a comparison of secondary outcomes in patients successfully managed by one single route as a face-to-face comparison of IPUS-AVP and CVC routes. A pre-specified analysis was performed according to stratification by number of leads to be implanted (single- or dual-chamber device).

Sample size calculation was based on a success rate of 95% in the IPUS-AVP group and 80% in the CVC group. These estimations were obtained from literature data.^9,11,20,22,25^ We calculated that 150 patients would be needed to show a 15% absolute difference in the success rate with a power of 80%. To ensure sufficient power, it was decided to enrol a total of 200 patients.

The normality of distribution of continuous variables was tested using the Shapiro–Wilk test. Continuous variables with normal distribution are presented as means ± standard deviation, and non-normally distributed variables are shown as medians with inter-quartile range or geometric means with 95% confidence interval (CI). Categorical variables are presented as counts and percentages. Endpoints were tested independently for differences between the two groups. Differences in categorical variables were assessed using the χ^2^ test or Fisher’s exact test, as appropriate. Continuous variables were compared using Student’s *t*-test or Mann–Whitney *U* test, as appropriate. Geometric mean ratios (GMRs) and two-sided 95% CI were calculated for positively skewed continuous variables by exponentiation of the mean difference of the logarithms and the corresponding confidence intervals (based on Student’s *t* distribution).

All tests were two-sided. All statistical analyses were performed using IBM SPSS Statistics for Windows version 26 (IBM Corp., Armonk, NY, USA).

## Results

### Trial participants

**Figure 2:**
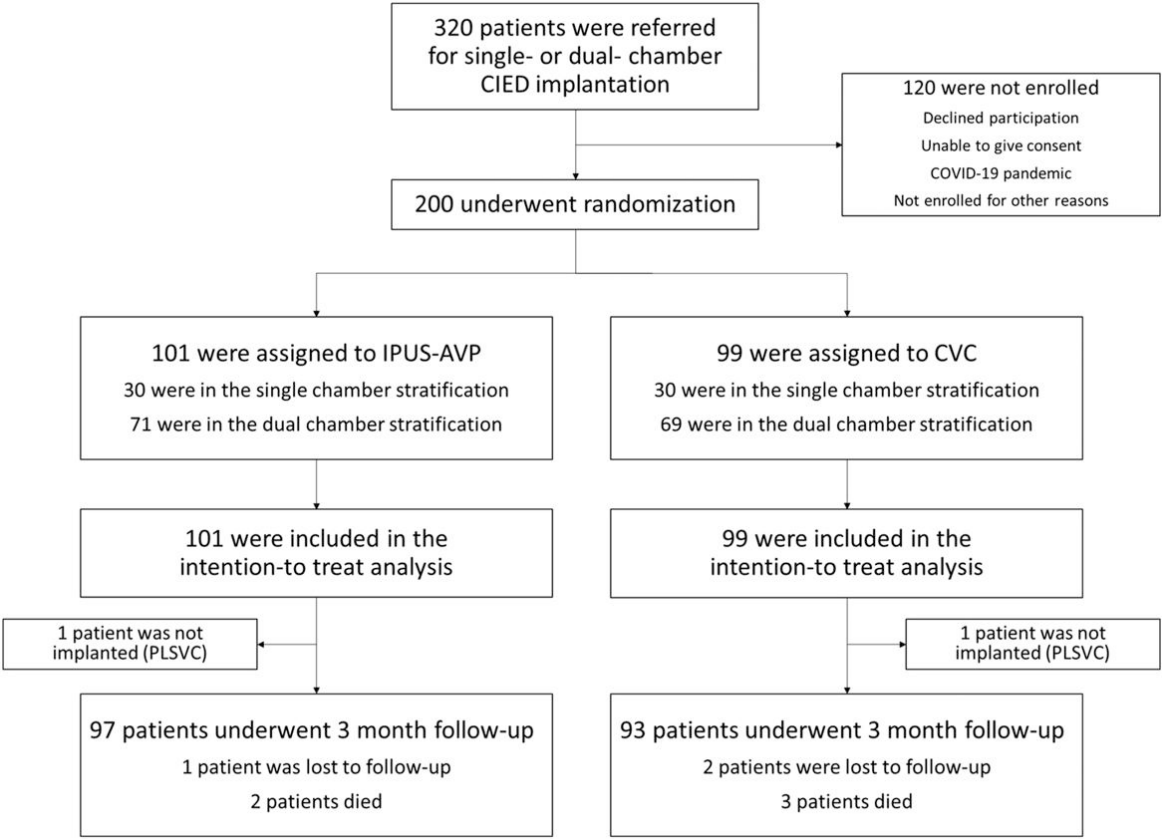
Study flowchart. CVC, cephalic vein cutdown; IPUS-AVP, intra-pocket ultrasound-guided axillary vein puncture; PLSVC, persistent left superior vena cava.

From the 320 patients eligible for the trial during the study period (July 2020–July 2022), 200 were finally included. Explanations for this inclusion rate mostly relied to the COVID-19 pandemic leading to shortage of clinical research staff and to an unexpected high rate of refusal (exacerbated by mistrust in health authorities during the pandemic) (see Supplementary data online, *Figure S1*); other reasons included the impossibility of obtaining consent from patients with cognitive impairment or language barriers. A total of 200 patients were included in the ITT analysis; 101 were randomized to IPUS-AVP, and 99 were randomized to CVC. Details regarding patient disposition are provided in *Figure 2*.

Patient characteristics at baseline are shown in *Table 1*. The characteristics of the two groups were not different. Mean age was 77 years with a male predominance (70.0%). Mean BMI was 26.0 kg/m². Antithrombotic therapy was prescribed in 139 (69.5%) patients. A total of 175 (87.5%) patients were referred for pacemaker implantation. The most frequent indication for CIED implantation was atrioventricular block (52.0% of patients). There were no clinically relevant differences in the characteristics of the non-included patients compared with the included patients (see Supplementary data online, *Table S1*).

**Table 1:** Baseline characteristics of the patients

	IPUS-AVP group (*n* = 101)	CVC group (*n* = 99)	*P*-value
Age—year	77 ± 11	78 ± 10	.67
Male sex—*n* (%)	70 (69.3)	70 (70.7)	.83
Female sex—*n* (%)	31 (30.7)	29 (29.3)	
Body mass index—kg/m²	25.6 ± 3.5	26.3 ± 4.6	.25
Hypertension—*n* (%)	60 (59.4)	65 (65.7)	.36
Diabetes—*n* (%)	29 (28.7)	36 (36.4)	.25
Concomitant cardiovascular conditions			
LVEF—%	65 (60–65)	61 (55–65)	.31
History of HFrEF—*n* (%)	10 (9.9)	12 (12.1)	.62
History of HFmrEF or HFpEF—*n* (%)	13 (12.9)	9 (9.1)	.39
Coronary artery disease—*n* (%)	27 (26.7)	37 (37.4)	.11
Valvular heart disease—*n* (%)	22 (22.0)	25 (25.8)	.53
Atrial fibrillation or flutter—*n* (%)	35 (34.7)	31 (31.3)	.62
Antithrombotic therapy—*n* (%)			.57
None	35 (34.6)	26 (26.3)	
Single antiplatelet therapy	24 (23.8)	32 (32.3)	
Dual antiplatelet therapy	7 (6.9)	5 (5.1)	
Anticoagulation alone	32 (31.7)	30 (30.3)	
Anticoagulation and mono APT	2 (2.0)	4 (4.0)	
Anticoagulation and dual APT	1 (1.0)	2 (2.0)	
Laboratory tests			
Creatinine—µmol/L	92 (75–116)	89 (73–107)	.76
PT—%	91 (81–98)	89 (75–97)	.31
Intervention			
Urgent—*n* (%)	61 (60.4)	63 (63.6)	.64
Indication—*n* (%)			.31
AV block	51 (50.5)	53 (53.6)	
Sinus node dysfunction	17 (16.8)	20 (20.2)	
Slow AF	14 (13.9)	11 (11.1)	
Tachycardia-bradycardia syndrome	7 (6.9)	1 (1.0)	
AV node ablation	0 (0.0)	1 (1.0)	
Prevention of SCD	12 (11.9)	13 (13.1)	
Number of leads to implant—*n* (%)			.93
Single chamber	30 (29.7)	30 (30.3)	
Dual chamber	71 (70.3)	69 (69.7)	

Data are mean ± standard deviation, median (inter-quartile range) or number (%). Data were missing on the following characteristics: LVEF for 11, valvular heart disease for 3 and PT for 4 patients.

ACT, activated clotting time; AF, atrial fibrillation; APT, antiplatelet therapy; AV, atrioventricular; CVC, cephalic vein cutdown; HFmrEF, heart failure with mid-range ejection fraction; HFpEF, heart failure with preserved ejection fraction; HFrEF, heart failure with reduced ejection fraction; IPUS-AVP, intra-pocket ultrasound-guided axillary vein puncture; LVEF, left ventricular ejection fraction; PT, prothrombin time; SCD, sudden cardiac death.

### Intervention

The procedural data are shown in *Table 2*. Two patients (one in each group) had an unpredictable impossibility of venous access due to an unknown persistent left SVC. For these two subjects, the primary outcome was considered a failure and the secondary outcomes were not assessed. Implantation was left-sided in 197 (98.5%) patients. A total of 335 leads were implanted with 61 single-chamber and 137 dual-chamber devices. Hydrophilic guidewire was used for cephalic vein access in 84 (84.8%) patients and for axillary vein access in two (2.0%) patients. Venous angiography was used for one patient (1.0%) in the IPUS-AVP group and two patients (2.0%) in the CVC group.

**Table 2:** Procedural data

	IPUS-AVP group (*N* = 101)	CVC group (*N* = 99)
Left-sided implantation—*n* (%)	101 (100)	96 (97.0)
Implanted device type—*n* (%)		
Pacemaker	88 (87.1)	85 (85.9)
ICD	12 (11.9)	13 (13.1)
None^a^	1 (1.0)	1 (1.0)
Number of implanted leads—*n* (%)		
Single chamber	30 (29.7)	31 (31.3)
Dual chamber	70 (69.3)	67 (67.7)
None^a^	1 (1.0)	1 (1.0)
Hydrophilic guidewire—*n* (%)	2 (2.0)	84 (84.8)
Venous angiography—*n* (%)	1 (1.0)	2 (2.0)

Data are number (%).

CVC, cephalic vein cutdown; IPUS-AVP, intra-pocket ultrasound-guided axillary vein puncture.

^a^Two patients (one in each group) were not implanted because of persistent left SVC.

Two patients scheduled to have a dual-chamber pacemaker received a single-chamber device because of chest pain and pericardial effusion after atrial lead placement. They did not require pericardial drainage. The atrial lead was extracted, and the procedure was successfully completed by implanting a single-chamber device. Both patients were in the CVC group. The operator’s decision to implant only a single-chamber device was not considered a procedural failure, as vascular access and lead insertion were successful.

### Endpoints

**Figure 3:**
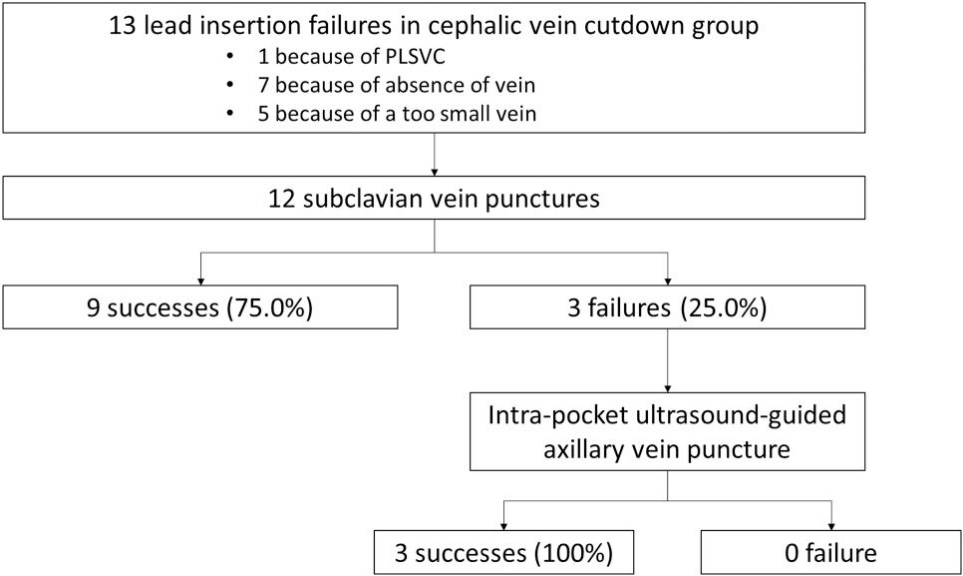
Description of procedural failures in the cephalic vein cutdown group. PLSVC, persistent left superior vena cava.

Primary and secondary endpoints are presented in *Table 3*. In an ITT analysis, the primary endpoint occurred in 100 patients (99.0%) in the IPUS-AVP group and in 86 patients (86.9%) in the CVC group (*P* = .001). As per protocol (*Figure 3*), the classical technique of CVC followed by SVP was successful in a total of 95 (96.0%) patients (*P* = .21 vs. IPUS-AVP). Secondary endpoints were assessed for the entire strategy (i.e. all the vascular access techniques) leading to a successful implantation of all leads. As compared to the CVC group, the IPUS-AVP group was characterized by a significantly reduced time to venous access [3.4 vs. 10.6 min, GMR 0.32 (95% CI 0.28–0.36)], time from venous access to skin closure [30.1 vs. 35.8 min, GMR 0.84 (95% CI 0.78–0.91)], and total procedure duration [33.8 vs. 46.9 min, GMR 0.72 (95% CI 0.67–0.78)] (*P* < .001 for all comparisons). Similarly, the fluoroscopy time and the X-ray exposure were significantly reduced in the IPUS-AVP group as compared to the CVC group [2.4 vs. 3.3 min, GMR 0.74 (95% CI 0.63–0.86) and 1083 vs. 1423 mGy.cm², GMR 0.76 (95% CI 0.62–0.93); *P* < .001 and *P* = .009, respectively].

**Table 3:** Primary and secondary endpoints

Primary endpoint (N = 200)	IPUS-AVP group (N = 101)	CVC group (*N* = 99)	Treatment effect^a^	*P*-value
Success—*n* (%)	100 (99.0)	86 (86.9)	1.14 (1.05–1.23)	.**001**
**Secondary endpoints** (*n* = 198)^b^	**IPUS-AVP group (*N* = 100)**	**CVC group (*N* = 98)**	**Treatment effect^c^**	***P*-value**
Time to venous access—min	3.4 (3.1–3.7)	10.6 (9.8–11.4)	0.32 (0.28–0.36)	**<**.**001**
Time from venous access to skin closure—min	30.1 (28.4–31.8)	35.8 (33.8–37.8)	0.84 (0.78–0.91)	**<**.**001**
Total procedure duration—min	33.8 (31.9–35.7)	46.9 (44.6–49.4)	0.72 (0.67–0.78)	**<**.**001**
Fluoroscopy time—min	2.4 (2.2–2.7)	3.3 (2.9–3.6)	0.74 (0.63–0.86)	**<**.**001**
X-ray exposure—mGy.cm²	1083 (937–1252)	1423 (1228–1649)	0.76 (0.62–0.93)	.**009**

Data are number (%) or geometric mean (95% CI). Bold values indicate statistical significance.

^a^Treatment effect is expressed as risk ratio (95% CI)

^b^Data for secondary endpoints were not assessed for 2 patients.

^c^Treatment effect is expressed as geometric mean ratio (95% CI).

CVC, cephalic vein cutdown; IPUS-AVP, intra-pocket ultrasound-guided axillary vein puncture.

A sensitivity analysis was performed in patients successfully managed by one single route (either IPUS-AVP or CVC) (see Supplementary data online, *Table S3*). Again, all secondary outcomes were significantly improved in the IPUS-AVP group as compared to the CVC group: time to venous access [3.4 vs. 10.1 min, GMR 0.33 (95% CI 0.29–0.38), *P* < .001], time from venous access to skin closure [30.1 vs. 35.0 min, GMR 0.86 (95% CI 0.79–0.93), *P* < .001], total procedure duration [33.8 vs. 45.7 min, GMR 0.74 (95% CI 0.68–0.80), *P* < .001], fluoroscopy time [2.4 vs. 3.1 min, GMR 0.77 (95% CI 0.66–0.91), *P* = .002], and X-ray exposure [1083 vs. 1349 mGy.cm², GMR 0.80 (95% CI 0.65–0.99), *P* = .041]. Sensitivity analyses were also performed excluding patients with a right-sided implantation, an ICD, or a removed atrial lead, with no change in the results (data not presented).

### Subgroup analyses

Endpoints stratified based upon single- and dual-chamber devices are presented in Supplementary data online, *Table S2*. As per the ITT principle, the two patients who received a single-chamber rather than the scheduled dual-chamber device were included in the dual-chamber subgroup. In the single-chamber subgroup, the primary endpoint occurred in 30 patients (100%) in the IPUS-AVP group and in 28 patients (93.3%) in the CVC group. In the dual-chamber subgroup, the primary endpoint occurred in 70 patients (98.6%) in the IPUS-AVP group and in 58 patients (84.1%) in the CVC group. Secondary endpoints were significantly improved with IPUS-AVP in both subgroups.

### Management of lead insertion failures

Thirteen failures (13.1%) occurred in the CVC group and one (1.0%) in the IPUS-AVP. Two failures (one in each group) were not related to vascular access technique, but to persistent left SVC. The management of lead insertion failures in the CVC group is presented in *Figure 3*. In the CVC group, 11 failures (84.6%) occurred for dual-chamber devices and only 2 (15.4%) for single-chamber devices. The causes of failure in the CVC group were persistent left SVC (*n* = 1; 7.7%), absence of vein (*n* = 7; 53.8%), vein too small for cannulation with the guidewire (*n* = 1; 7.7%), and vein too small for insertion of two leads (*n* = 4; 30.8%). Subclavian vein puncture was performed in second line successfully in nine patients (75.0%). In three subjects (25.0%), the operators switched from SVP to IPUS-AVP after several failed attempts, as allowed by the protocol, in order to avoid complications in frail patients. Intra-pocket ultrasound-guided axillary vein puncture was successful in all cases.

### Safety and complications

A total of 190 patients (95.0%) completed the full 3-month follow-up after the procedure (*Figure 2*). Three patients were lost to follow-up (one in the IPUS-AVP group and two in the CVC group). Five patients died (two in the IPUS-AVP group and three in the CVC group). These deaths were unrelated to the procedure.

There were a total of five complications (2.5%) (*Table 4*). There was no significant difference in complication rates between the two groups (*P* = .68). In the IPUS-AVP group, there was one pocket infection and one lead dislodgment. In the CVC group, there were two pericardial effusions and one lead dislodgment. The pocket infection occurred in a patient at higher risk of infection (87 years old, diabetic, and suffering from rheumatoid arthritis). The patient was successfully treated with antibiotic treatment, device extraction and leadless pacemaker implantation. The two pericardial effusions occurred in elderly females (81 and 88 years old), who both experienced chest pain during the screwing of the atrial lead. Ultrasonography showed a mild pericardial effusion without cardiac tamponade, which did not require pericardiocentesis. A single-chamber device was finally implanted, and the patients were monitored for a few more days before discharge. There was no major pocket haematoma, pneumothorax, haemothorax, brachial plexus palsy, or deep vein thrombosis reported in this study.

**Table 4:** Procedural and post-operative complications

	IPUS-AVP group (*N* = 101)	CVC group (*N* = 99)
Device infection	1 (1.0)	0
Lead dislodgment	1 (1.0)	1 (1.0)
Pericardial effusion	0	2 (2.0)
Tamponade	0	0
Pneumothorax	0	0
Pocket haematoma	0	0
Total	2 (2.0)	3 (3.0)

Data are number (%).

CVC, cephalic vein cutdown; IPUS-AVP, intra-pocket ultrasound-guided axillary vein puncture.

## Discussion

The results of this randomized controlled clinical trial indicate that IPUS-AVP is superior to CVC in terms of success rate, time to venous access, procedure duration, and radiation exposure. Moreover, the rate of complications was not significantly different between the groups (Structured Graphical Abstract).

Intra-pocket ultrasound-guided axillary vein puncture is little described in the literature^18,20–22^ and, to the best of our knowledge, our study is the first randomized trial to evaluate this technique. Our trial is also the largest to date to investigate AVP with US guidance for CIED implantation.

The CVC technique was chosen for the control arm because it is the most widely used technique in Europe (first intention for 60% of operators).^5^ The study protocol clearly specified the technique to use in case of failure in each group. The protocol was in accordance with the ESC guidelines, which recommend avoiding SVP in first line.^2,4^ The randomization with stratification based on the number of leads to be implanted allowed an unbiased comparison with CVC. The sample size calculation provided enough power to detect a statistically significant difference between the two groups for all endpoints. There were few inclusion and exclusion criteria, which provide good external validity. Finally, patients were monitored for 3 months after operation, with few patients lost to follow-up.

The success rate of IPUS-AVP alone was 99.0% and the only failure was not related to the vascular access but to the anatomy (persistent left SVC). For comparison, the two randomized trials that investigated US-AVP with a percutaneous technique, by Liccardo *et al.*^19^ and Tagliari *et al.*,^20^ reported a success rate of 91.4% and 97.7%, respectively. The high success rate in our study can be explained by several reasons. First, the primary operators involved in this study were all senior physicians well experienced with this technique. Second, the dissection of the subcutaneous tissue before the puncture allowed the US probe to be directly in contact with the pectoral muscle, which improves echogenicity and visualization of the axillary vessels. Third, the use of an L-shaped ‘hockey stick’ US probe that is small and easy to handle in the wound, compared to a classical linear vascular transducer, also contributed to improve image quality. This is a very important point, as this type of probe is essential to easily puncture inside the pocket. The use of this probe with an IPUS-AVP technique resulted in very good visualization of the vein and needle path, regardless of patient’s anatomy. This is a major advantage over percutaneous puncture which often fails in patients with a high BMI or a deeper vein.^26,27^ Moreover, our team also routinely uses intravenous volume expansion and Trendelenburg position to enlarge the vein if it is small or difficult to visualize.

In the CVC group, the success rate of CVC route alone was higher than expected, with 86.9% of cases. The level of experience of the operators and the almost systematic use of hydrophilic guidewire account for part of these numbers. The high success rate for CVC observed in this study may also be explained by our definition of success. To reproduce the conditions observed in real-life clinical practice, it was deliberately decided not to set a limit on duration of procedure or the number of attempts to define a failure. It should be pointed out that, although impossibility to blind the operator to treatment allocation could have biased endpoints, especially in the CVC group, success rates and time to obtain venous access are better than those reported in previous randomized trials. In particular for CVC, Chan *et al.* reported a 78.2% success rate, Squara *et al.* reported a 75.7% success rate and an average time of 12.2 min to obtain venous access, Jiménez-Díaz *et al.* reported a 76.7% success rate and an average time of 13.1 min, Tagliari *et al.* reported a 54.5% success rate (with a time limit of 15 min) and an average time of 15 min.^9–11,20^

The cephalic vein is sometimes too small, very tortuous, or even absent, whereas the axillary vein has few anatomical variations and is large enough to accommodate multiple leads.^8,11,20^ In this study, 12 patients underwent SVP in second line, as specified by the protocol. This second-line technique was difficult in three patients because of a particular anatomy. In these cases, IPUS-AVP was easily successful and avoided the risks of repeated blind punctures, which likely explains the absence of pneumothorax in our study. It could therefore be useful to have the possibility of performing IPUS-AVP in difficult cases.

Reduction of time to venous access with IPUS-AVP was expected, as CVC requires time-consuming surgical dissection^6–11^; this was confirmed by a direct comparison performed in a subset of patients successfully managed by one single route (either IPUS-AVP or CVC). Albeit being equally successful in terms of leads implantation, the frequent need of the CVC-SVP combination further increases the duration of the time to venous access in this group. Interestingly, we observed not only a decrease in vascular access time but also a reduction in the time between vascular access and skin closure. This difference is probably explained by greater ease of lead manipulation during its placement and fixation (especially the absence of rubbing between the leads). Also, the systematic use of separate punctures (instead of a retained guidewire technique) for the implantation of dual-chamber devices limits bleeding (by restricting the size of the perforation) and therefore the time dedicated to haemostasis. All these advantages result in a reduction of total procedure duration with IPUS-AVP. Procedures are thus faster, with less variability in duration, which reduces operator fatigue and increases the number of operations that can be performed daily.

Our study also showed a significant reduction in fluoroscopy time and X-ray exposure with IPUS-AVP, again confirmed by the sensitivity analyses performed in those patients successfully managed by a single route. One of the causes of this decrease is the easier insertion of the guidewire in the SVC with IPUS-AVP, without need for fluoroscopy in most cases. Indeed, the path of the cephalic vein is often tortuous, so that fluoroscopy guidance is frequently needed in the CVC technique to navigate the guidewire to the SVC. The reduction in the use of fluoroscopy with IPUS-AVP is also explained by easier lead manipulation and improved stability. Even with relatively low radiation doses, this reduction is an important element of protection against radiation for daily exposed operators. Fluoroscopy usage was not evaluated in the previously mentioned trials.^19,20^

Complications rates were comparable between the two groups. No pneumothorax occurred in our study, which is consistent with previous reports of a low risk of pneumothorax with fluoroscopy- or US-guided AVP.^19,20^ This risk becomes almost non-existent with IPUS-AVP because the needle tip is clearly visible along its path from the skin to the vein, which prevents accidental pleural puncture. We did not observe any major pocket haematoma in our study even though 69.5% of patients were treated with antithrombotic therapy, which is usual among patients implanted with CIED. This low incidence is consistent with recent data on pocket haematoma after CIED implantation in patients treated with direct oral anticoagulants.^28^ Haemostasis with IPUS-AVP is therefore as effective as with CVC. As discussed previously, in case of multiple lead insertion, we recommend performing separate punctures to limit the size of the puncture holes and the bleeding. The incidence of iatrogenic cardiac perforation is coherent with published data.^29^ One pocket infection occurred in the IPUS-AVP group. It should be highlighted that the use of a non-sterile material such as the US probe requires vigilance against aseptic errors when placing it in the sterile sheath. Procedure duration, on the other hand, is a well-known infectious risk factor,^30,31^ so that its reduction should reduce the occurrence of infections. Unfortunately, our study was not sufficiently powered to assess this risk. A large multi-centric trial would be needed to confirm the safety of this procedure.

### Limitations

Our study has some limitations. First, it is monocentric and has few operators. However, the purpose of this study was to evaluate the technic as performed by adequately trained operators so as to minimize a potential negative bias incurred by a training deficit. The results therefore apply to experienced operators using the same materials as in this study. Nevertheless, this technique is easy to learn. Second, the duration of follow-up cannot detect late complications, particularly lead dysfunction, but previous studies on fluoroscopy-guided AVP described a low incidence for this complication.^1,32^ Third, our study did not investigate lead revision, upgrading, and biventricular procedures. In our daily practice, US guidance is also very effective for these interventions, but this should be tested in another study. Finally, from the 320 patients eligible for the trial during the study period, only 200 were included mostly because of COVID-19 pandemic. However, the characteristics of the non-included patients are similar to those of the included patients (see Supplementary data online, *Table S1*).

### Clinical implications

Most electrophysiology laboratories have access to an US machine, as transoesophageal echocardiography is widely used for identifying atrial thrombus and guiding trans-septal puncture during electrophysiology procedures.^33^ This positive trend should continue as US-guided femoral approach has demonstrated a decrease in complications.^34^ Thus, the barrier to the use of US guidance for CIED implantation does not come from a lack of equipment but rather from a lack of perceived benefit or absence of adequate operator training. However, IPUS-AVP is easily accessible to anyone familiar with the practice of echography, which is usually the case for cardiologists. For operators experienced to CIED implant but new to US guidance, the learning curve for improving procedural times over CVC was estimated to be between 15 and 25 procedures.^23,25,35^

Uncertainty remains about the best vascular access strategy. It is now well established that subclavian vein is associated with more complications.^1,4^ IPUS-AVP can therefore be used as a second-choice technique in case of failure with CVC, in order to avoid SVP. IPUS-AVP can also be used in first intention, as proposed in this study. The design of our trial does not enable to conclude which strategy is the best between percutaneous and intra-pocket US-AVP. A dedicated multi-centric study would be needed to assess this specific question. In any case, operators should be familiar with several techniques to manage every challenging situation.

## Conclusion

Intra-pocket ultrasound-guided axillary vein puncture is superior to CVC in terms of success rate, time to venous access, procedure duration, and radiation exposure, with a similarly low complication rate. This technique significantly improves vascular access, which is the main challenge and source of complications in CIED implantation, by making it easier and more reproducible. Intra-pocket ultrasound-guided axillary vein puncture improves the quality of care and should therefore be included in the recommended venous access techniques for CIED implantation.

## Supplementary Material

ehad629_Supplementary_Data

## Data Availability

The data that support the findings of this study are available from the corresponding author upon reasonable request.

## Pre-registered Clinical Trial Number

The pre-registered clinical trial number is NCT04649788.
