# Supplementary material for: Intra-pocket ultrasound-guided axillary vein puncture vs. cephalic vein cutdown for cardiac electronic device implantation: the ACCESS trial
Source: Eur Heart J. 2023 Oct 13;44(46):4847–58. doi: 10.1093/eurheartj/ehad629 (PMC10702459; doi:10.1093/eurheartj/ehad629)
Supplement: ehad629_Supplementary_Data [file ehad629_supplementary_data.zip › Supplementary Legends.docx]

**SUPPLEMENTARY LEGENDS**

**Supplementary data Figure S1** – Patients inclusions and COVID-19 pandemic waves.

**Supplementary data Video 1** – Video illustration of the intra-pocket ultrasound-guided axillary vein puncture technique.

IPUS-AVP = intra-pocket ultrasound-guided axillary vein puncture; US = ultrasound.
