# Supplementary figures and images for: Intra-pocket ultrasound-guided axillary vein puncture vs. cephalic vein cutdown for cardiac electronic device implantation: the ACCESS trial
Source: Eur Heart J. 2023 Oct 13;44(46):4847–58. doi: 10.1093/eurheartj/ehad629 (PMC10702459; doi:10.1093/eurheartj/ehad629)

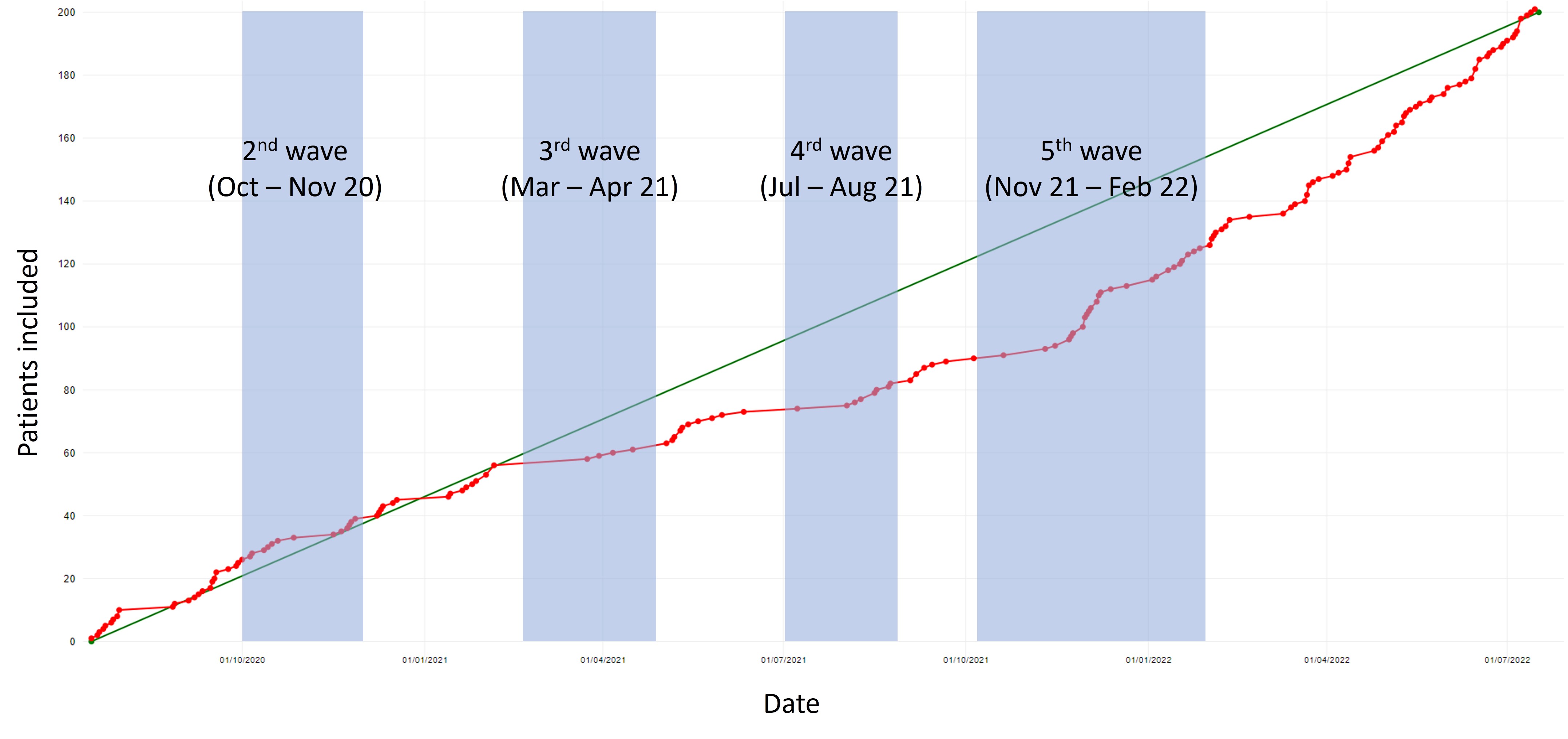

Supplement: ehad629_Supplementary_Data [file ehad629_supplementary_data.zip › Supplementary Figure S1.jpg]
